# Latency Trend Analysis as a Guide to Screening Malignancy Survivors for Second Primary Thyroid Cancer

**DOI:** 10.3390/biomedicines10081984

**Published:** 2022-08-16

**Authors:** Mohammad Hussein, Lauren Mueller, Peter P. Issa, Muhib Haidari, Lily Trinh, Eman Toraih, Emad Kandil

**Affiliations:** 1 Department of Surgery, School of Medicine, Tulane University, New Orleans, LA 70112, USA; 2 School of Medicine, Tulane University, New Orleans, LA 70112, USA; 3 School of Medicine, Louisiana State University Health Sciences Center, New Orleans, LA 70112, USA; 4 Division of Thyroid and Parathyroid Endocrine Surgery, Department of Otolaryngology-Head and Neck Surgery, Massachusetts Eye and Ear Infirmary, Harvard Medical School, Boston, MA 02114, USA; 5 Genetics Unit, Department of Histology and Cell Biology, Faculty of Medicine, Suez Canal University, Ismailia 41522, Egypt

**Keywords:** thyroid latency, second primary thyroid cancer, SEER, screening

## Abstract

Primary cancer survivors have a higher risk of developing second primary thyroid cancer (SPTC). Patients with SPTC who survived primary malignancies, diagnosed from 1975 to 2016, were identified from the Surveillance, Epidemiology, and End Results (SEER) database (SEER 18 Registry). A total of 33,551 cancer cases were enrolled in the final analysis. Individuals with a primary malignancy were at a significant 90% increased risk of developing SPTC (SIR = 1.90, 95%CI = 1.86–1.93, *p* < 0.05) compared to the general population. More than half (54.7%) of SPTC diagnoses were made in the first three years after primary cancer diagnosis, and the most aggressive presentations of SPTC occurred within the first year following malignancy. A latency trend analysis identified persistent high risk for development of SPTC after diagnosis of lymphoma, leukemia, soft tissue tumors, kidney, breast, and uterine cancer; elevated 10-year risk for most cancers such as salivary gland, melanoma, stomach, lung, colon, ovarian, pancreas, prostate, and bladder; and high 5-year risk after cancers such as larynx, oral, orbit, bone, small intestine, and liver. Our latency period model identifying risk according to each type of primary cancer may aid clinicians in identifying at-risk patients to be screened for thyroid cancer and guide them in developing a surveillance plan according to the latency period attributed to a patient’s primary cancer.

## 1. Introduction

Thyroid cancer (TC) is currently the most common endocrine malignancy in the United States [1]. Over the past several decades, the incidence and mortality of TC has increased significantly [2,3]. The American Cancer Society estimates there to be 43,800 new TC cases (11,860 in men and 31,940 in women) and 2230 TC deaths (1070 men and 1160 women) in the United States for the year 2022 alone [4].

There are various proposed causes for the increased TC incidence, including overdiagnosis secondary to recently available advanced diagnostic tools and radiation cancer treatments [3,5]. To investigate the latter cause and its associated increase in TC incidence, researchers have investigated the correlation between a primary malignancy and the later development of TC, termed second primary thyroid cancer (SPTC). The British Childhood Cancer Survivor Study reported an increased risk of TC after Hodgkin disease and non-Hodgkin lymphoma [6]. Lal et al. demonstrated that the risk of TC as a second primary neoplasm was greatest for renal and breast cancer survivors [5]. In some studies, the elevated risk was attributed to receiving radiotherapy for the treatment of the primary cancer, however others report an increased risk in patients who did not undergo radiation treatment [5,6,7,8]. Previous studies discuss the overall risks of cancer development without exploring the latency trends beyond the first year of initial diagnosis or confine their work to a single type of TC [9]. Lal et al. analyzed a sample population that includes only nine distinct geographical regions in the United States from 1975 through 2008, a timeframe that predates the current management strategies and guidelines [5]. Additionally, factors such as histological subtype, clinical presentation, and previous cancer treatment have not been addressed in the context of SPTC.

The objective of this study is to evaluate the risk of SPTC in primary cancer survivors. Our study uniquely includes the largest sample size with the most types of cancers analyzed in the literature thus far. We also evaluated whether these risks were associated with specific TC histological subtypes, clinical presentation, and previous cancer treatment. The broader geographic coverage and larger sample size in this current study will improve the generalizability of the risk factors and latency period of developing SPTC. Physicians can utilize our findings to identify at-risk patients to screen for TC and develop a surveillance plan for cancer survivors.

## 2. Materials and Methods

### 2.1. Study Sample

A population-based cohort was identified from the National Cancer Institute’s Surveillance, Epidemiology, and End Results (SEER) database. The SEER Program publishes cancer incidence and survival data from cancer registries covering approximately 30% of the US population [10]. The study included cancer patients from 18 different geographical regions from 1975–2016 (San Francisco-Oakland SMSA, Connecticut, Detroit (Metropolitan), Hawaii, Iowa, New Mexico, Seattle (Puget Sound), Utah, Atlanta (Metropolitan), San Jose-Monterey, Los Angeles, Alaska Natives, Rural Georgia, California excluding SF/SJM/LA, Kentucky, Louisiana, New Jersey, Greater Georgia). The SEER data is publicly available, and, in consequence, the study was exempt from Institutional Review Board approval. We signed the Research Data Agreement before this study and obtained access to the database with the username 15332-Nov2019.

Patients were included if they presented with TC as their second or higher cancer. According to the World Health Organization (WHO), cases diagnosed within two months of the initial primary tumor were considered synchronous cancers and were excluded. Patients with primary malignancies in the thyroid were excluded. Though increased medical surveillance would increase the detection of incidental SPTCs (as it would in all subsequent primary cancer studies), we excluded Death certificates or autopsy-only patients in an attempt to limit the analysis to clinically relevant tumors.

### 2.2. Cohort Extraction

Cases of primary malignancies were extracted from the SEER 18 registry (1975–2016) using SEER∗Stat software (version 8.3.6; Surveillance Research Program, National Cancer Institute, Bethesda, MD, USA; www.seer.cancer.gov/seerstat: accessed date: 15 January 2021) and imported into IBM Statistical Package for the Social Sciences (SPSS) version 27.0 (IBM Corp., Armonk, NY, USA). Identified cases of malignancies were then analyzed for standardized incidence ratios (SIR) and absolute excess risks (AER) of subsequent TC. International Classification of Diseases for Oncology (ICD-O-3) was adopted to identify the cancer site (thyroid) and subtype, classified based on the WHO classification of TCs [11]. For these groupings, the follicular variant of papillary TC was included with the papillary thyroid carcinoma group due to their similar clinical behavior. Both adult (>18 years) and pediatric cohorts were included to identify heterogeneous patterns in both age groups.

### 2.3. Variables and Outcomes

The incidence of second TC in primary cancer survivors was compared to the general population in the United States. Variables and outcomes were analyzed in a similar manner to previously published work in the field [9,12]. The latency period was defined as the time duration from which the first primary cancer was diagnosed until the date of either the index primary TC diagnosis, death, or the end date of the study, which was 31 December 2016, whichever occurred first. The observed number and the expected number of SPTC were identified. For each type of primary cancer, the relative risk for SPTC compared to the general population in the 18 SEER areas was presented as SIR along with its 95% confidence interval (CI).

The number of expected cancers was calculated based on five-year age-specific and sex-specific cancer incidence rates by cancer type for the general population. The number of person-years at risk was stratified by latency periods after two months following primary cancer diagnosis (2–11 months, 12–59 months, 60–119 months, and 120+ months). Person-years at risk represented the number of people with cancer per year at risk of developing TC. Cancer incidence rates were multiplied by the corresponding person-years at risk to estimate the number of cancer cases expected in each stratum. SIRs were calculated as the ratio of observed numbers to expected numbers. As aforementioned, the number of expected TC was estimated using 5-year age-specific and sex-specific cancer incidence rates for the general population. The AER was calculated by subtracting the expected from the observed number of patients with SPTC, dividing the difference by person-years at risk, and multiplying by 10,000. This number measures the overall burden due to the second primary cancer.

To investigate the molecular profile of primary tumors and their relation to SPTC, we used the Gene Expression Profiling Interactive Analysis (GEPIA) database (http://gepia.cancer-pku.cn/; accessed on 7 August 2022) to analyze the RNA sequencing expression of 5285 tumor and 3135 normal samples from the Genotype Tissue Expression project (GTEx) and The Cancer Genome Atlas (TCGA) [13].

### 2.4. Statistical Analysis

Multiple Primary-SIR programs (version 8.3.6, SEER Program, National Cancer Institute, Bethesda, MD, USA) in the SEER*Stat software package was used to obtain SIRs and AERs of subsequent TC. To compare the SIR of patients with different clinicopathological features, significance (*p*-value) was identified by the estimated z-score. *p*-values of <0.05 were considered statistically significant. All statistical tests were two-sided.

## 3. Results

### 3.1. Study Population

Data for a total of 7,586,281 records with cancer were reviewed. 33,551 cancer cases linked to 15,620 SPTC individuals (women: n = 9730, 62.3%; men: n = 5833, 37.7%) were enrolled in the final analysis. Patients aged 45–85 years constituted the majority of the study population (85.6%). Of the 33,551 cancers analyzed, 219 were found to be in patients younger than 20 years of age and only 70 were found in patients younger than 10 years of age. Of the SPTC patients, 13,980 (89.5%) had one previous primary cancer, while 1443 (9.2%) and 197 (1.3%) had two or more primary tumors preceding TC, respectively. Characteristics of the patients are summarized in Supplementary Table S1.

### 3.2. Incidence of Thyroid Cancer

**Figure 1:**
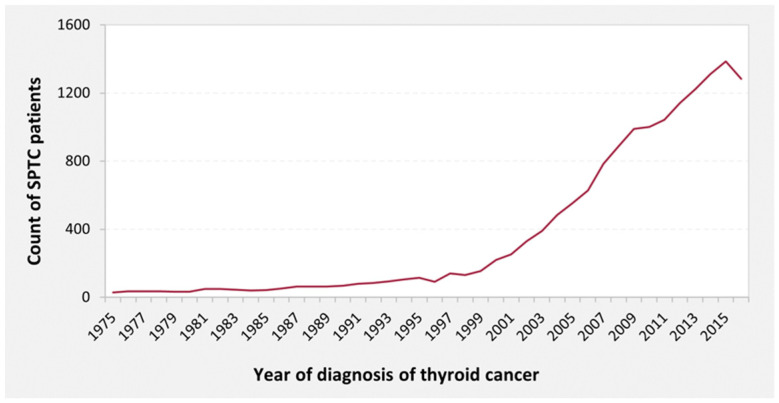
The annual increase in diagnosed cases of thyroid cancer. SPTC: Second primary thyroid cancer.

Since 1997, the incidence of SPTC has increased an average of 5% annually, reaching an eightfold increase in 2015 (Figure 1). Incidence increased more dramatically in females across the past two decades, especially in patients aged 45–75 years.

### 3.3. Spatial Analysis of Primary Cancer Preceding SPTC

**Figure 2:**
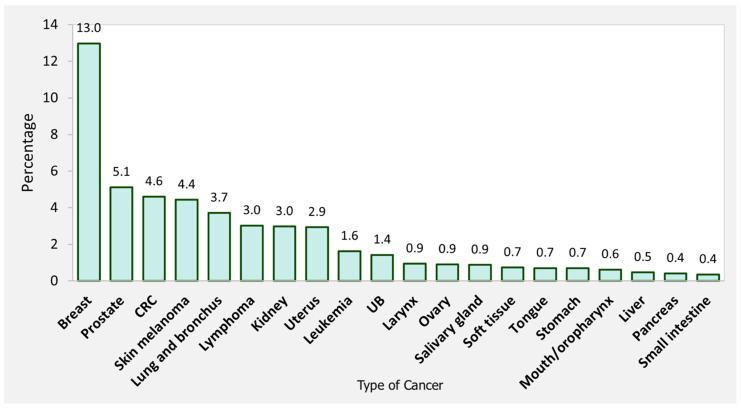
Type of primary cancer as a percentage of patients with primary malignancy developing subsequent thyroid cancer. UB: Urinary and bladder cancer, CRC: Colorectal cancer.

The most frequent primary cancers were breast (n = 4354, 13%), prostate (n = 1718, 5.1%), colorectal (n = 1545, 4.6%), skin melanoma (n = 1487, 4.4%), lung and bronchus (n = 1247, 3.7%), lymphoma (n = 1015, 3.0%), renal (n = 998, 3.0%), uterus (n = 984, 2.9%), leukemia (n = 545, 1.6%), and bladder cancers (n = 478, 1.4%; Figure 2).

### 3.4. Standardized Incidence Ratios for SPTC

Overall analysis showed a significant 90% increased risk of SPTC (SIR = 1.90, 95%CI = 1.86–1.93, *p* < 0.05) in primary cancer patients compared with the general population. The risk of developing SPTC varies among different cancer sites. The most significant relative risks of developing SPTC were observed following soft tissue sarcomas (SIR = 4.51, mean age = 53.9 years), salivary gland tumors (SIR = 4.36, mean age = 55.2 years), extranodal Hodgkin lymphomas (SIR = 4.25, mean age = 50.8 years), followed by malignancies in the nasal cavity and middle ear (SIR = 4.12, mean age = 61.8 years), mesothelioma (SIR = 3.9, mean age = 65.8 years), tongue (SIR = 3.8, mean age = 59.4 years), and kidneys (SIR = 3.8, mean age = 61.5 years). Cancers of the lip, penis, hepatic and splenic flexures, ureters, biliary tract, and gallbladder were not associated with increased overall SIR for SPTC (Table 1).

### 3.5. Temporal Analysis for the Latency Period to Develop SPTC

**Figure 3:**
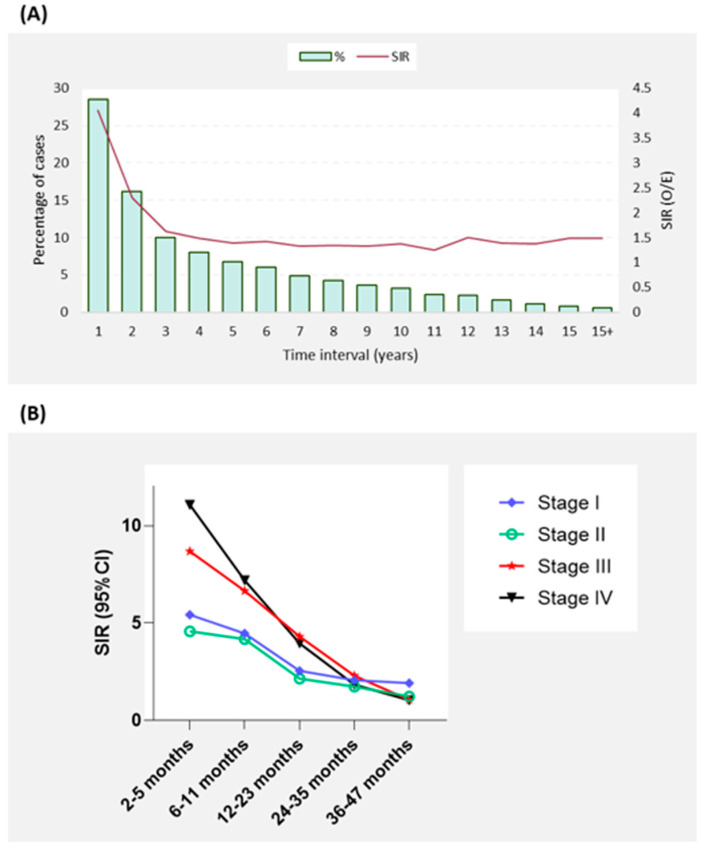
Overall incidence and risk of SPTC overtime after a primary cancer diagnosis. First-year does not include the first two months. SPTC: Second primary thyroid cancer. SIR: standardized incidence ratio (observed/expected). (**A**) Annual percentage of patients developing SPTC. (**B**) Latency course of SPTC development stratified by TC stage.

**Figure 4:**
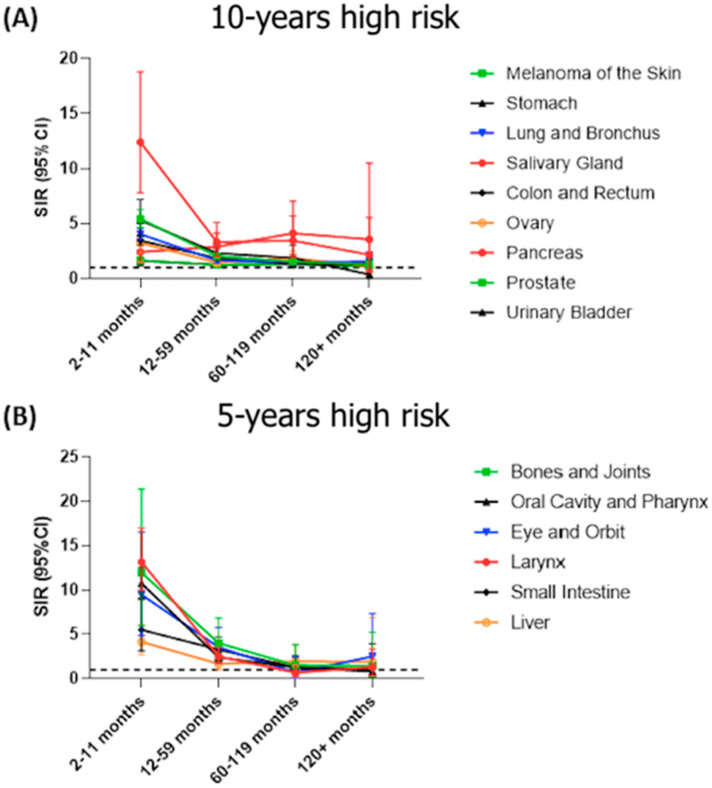
Latency course of elevated SIRs of developing second primary thyroid cancer risk in patients according to selected primary cancer. Primary cancers which mantained increased risk at (**A**) 10-years and at (**B**) 5-years. SIR: standardized incidence ratio (observed/expected), CI: confidence interval.

**Figure 5:**
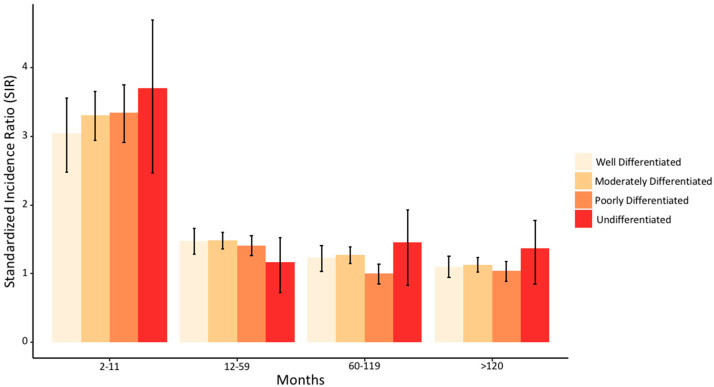
Risk of developing SPTC stratified by tumor differentiation status over time. SIR: standardized incidence ratio (observed/expected).

The total observation period was 23,988,205 person-years with a mean of 5.02 person-years at risk. Mean age of SPTC development was at 61.5 years. A quarter of SPTC cases developed in the first year following primary cancer diagnosis with the highest elevated risk (SIR = 4.06, 95%CI = 3.91–4.22, *p* < 0.05). These cases which developed within the first year were also the most aggressive with the most advanced cancer staging (Figure 3B). Overall, the first three years after primary cancer diagnosis are the most critical to screen for TC as over half (54.7%) of SPTC cases are diagnosed within this period (Figure 3A). To aid clinicians in identifying risk for their patients, we examined associated SIRs and latency patterns according to each specific primary cancer. Primary cancers in soft tissue, kidney, leukemia, breast, uterus, and lymphoma had persistently high SIRs for SPTC. The risk was more evident within the first 10 years of follow-up for most cancer sites, including salivary glands, skin melanoma, stomach, colorectal, pancreas, lung, ovary, prostate, and urinary bladder (Figure 4A). SIRs for SPTC were also elevated during the first five years after initial diagnosis of larynx, oral cavity, eye and orbit, bone, small intestine, and liver cancer, after which elevated risk was normalized (Figure 4B). In general, tumors did not differ with respect to differentiation classification with time (Figure 5). Detailed information about the SIRs and latency periods for each type of cancer is demonstrated in Supplementary Table S2.

### 3.6. Risk of SPTC According to Patient Demographics and Primary Cancer Characteristics

**Figure 6:**
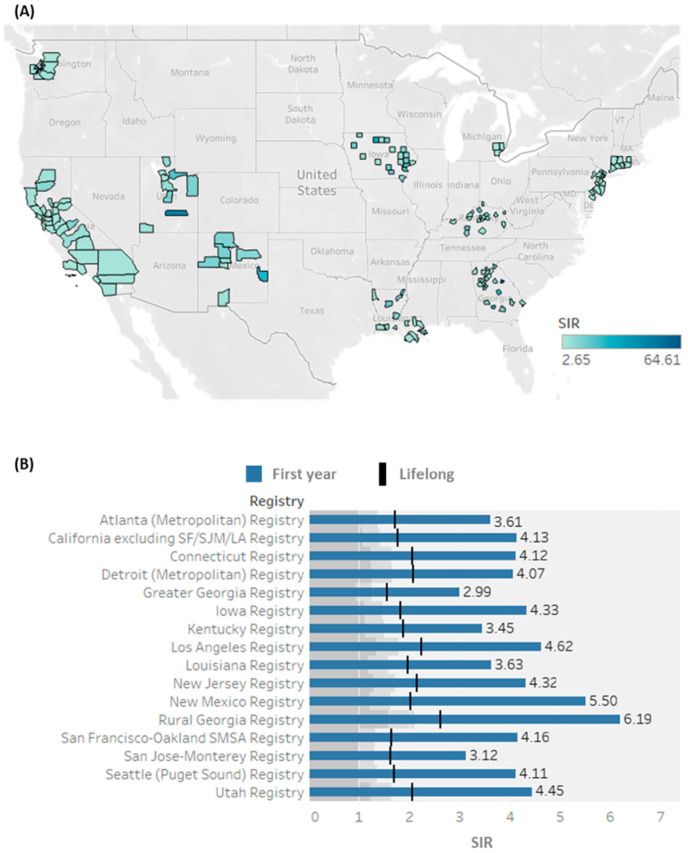
Geographical distribution of elevated risk developing second primary thyroid cancer (SPTC) in the United States. SIR: standardized incidence ratio (observed/expected). (**A**) Mapping counties with significant elevated SIR (n = 155 counties) during the first year following another primary malignancy. Color shows SIR for county, state, and zip code. (**B**) SIR across SEER registries during the first year and lifelong period.

As depicted in Table 2, there was a significantly higher risk of SPTC in males compared to females (*p* < 0.001) and in Asian/Pacific Islander populations compared to White Caucasians (*p* < 0.001). Across 630 counties in SEER registry 18, residents in 155 counties were significantly at-risk of SPTC in the first year following primary cancer (Figure 6). Midline tumors, observed in 18 patients, also had a significantly higher risk compared to those with tumors in unpaired or bilateral organs (*p* = 0.009). Despite the increased SIR in patients receiving different therapeutic modalities, a significantly higher risk of SPTC was primarily seen in patients who received chemotherapy than those having surgery at primary sites (*p* < 0.001). Compared to those who did not receive chemotherapy, higher SIRs were noted with chemotherapy following malignancies of bone (SIR = 7.33 vs. 2.43), soft tissue (6.36 vs. 4.13), oropharynx (4.41 vs. 1.11), hypopharynx (3.68 vs. 2.7), colon (2.22 vs. 1.60), stomach (2.30 vs. 1.60), uterus (2.73 vs. 1.60), and prostate (2.68 vs. 1.30). In contrast, patients receiving neoadjuvant treatment showed lower risk in pancreatic (2.05 vs. 3.92) and ovarian (1.63 vs. 2.18) tumor development than those not treated with chemotherapy. SIRs in patients treated with different therapeutic modalities are demonstrated in Table 3.

### 3.7. The Overall Mortality of SPTC

Of the 1828 (32.6%) SPTC patients who died, cancer-related deaths accounted for 16.7%. Top causes of death were primary cancers in the lung (n = 305, 16.7%), breast (n = 244, 13.3%), and colon (n = 126, 6.9%). For those with multiple primary cancers, the frequency of overall mortality was significantly higher (38.9% for two primary cancers and 47.2% for three or more primary cancers) than for a single tumor (31.4%). Increased infiltration, metastasis, and staging were associated with two or more primary cancers, indicating an increase in aggressiveness on presentation.

## 4. Discussion

To our best knowledge, this present study is the largest and most comprehensive database analysis of primary cancer survivors with subsequent SPTC. This study analyzes long and short-term latency trends and stratifies each risk by type of primary malignancy, contributing additional insight to previous literature [9,12,14,15,16]. Our study is also the first to examine risks pertaining to tumor grade, laterality, and metastasis.

According to our results, primary cancer survivors are at a 90% increased risk of developing SPTC than the overall population, which is higher than the reported risk in previous literature. In 2005, Ronckers et al. reported a 42% higher risk of SPTC than the standard population [17]. This discrepancy may be explained by a significantly smaller sample size of 1366 compared to this present study of 15,620. It should also be noted that the previous work analyzed an earlier timeline (1973 to 2000), and according to our analysis, the incidence of SPTC has increased by at least 5% annually since 1997. Similarly, the work of Schoenfeld et al. reported a 50% increased risk of developing second primary papillary TC [9]. Importantly, as is the case with all subsequent primary cancer detection studies, SPTC detection rates are expected to be slightly artificially inflated considering increased medical surveillance in consequence of treatment of the primary cancer.

Increased head and neck area surveillance, such as follow-up ultrasounds, may account for elevated SIRs of TC. While treatment of past head and neck cancer near the thyroid may influence SPTC development, previous literature has excluded radiation and chemotherapy as sole causative factors [5,18]. The latency period of radiation therapy is 20–30 years [19]. We observed a mean of 5.02 person-years at risk, excluding radiation treatment as the sole indicator of increased risk. Radiation therapy to treat a hematopoietic malignancy in children has consistently proven to be associated with an increased risk of developing a subsequent malignancy, specifically TC [7,20,21,22]. Interestingly however, radiotherapy is not associated with increased papillary TC development in ≥5-year cancer survivors with previous primary malignancy near and/or around the neck treated by radiotherapy [9]. With respect to chemotherapy, we found an increased risk of SPTC following treatment. However, prior reports suggest that it contributes an additive effect to radiation rather than an independent causative effect [7]. In our study, the median age for the development of SPTC was younger than the typical onset of TC in childhood cancers such as acute lymphocytic leukemia (ALL), Hodgkin lymphoma, and brain cancer. Compared to a multinational estimate (cases from Europe, Australia, and Canada), development of SPTC at an age younger than 65 was reported in 45.8% of cases [16]. In our United States-based population, we found that 54.4% of cases occurred before the age of 65, suggesting that SPTCs arise earlier in our population. 

A further possible explanation of the increased risk of developing SPTC after specific primary malignancies may be associated with similar genetic susceptibility for cancer development. For example, there is an increased risk of breast, prostate, and TC with RET gene mutation [23,24]). MDM2 *rs2279744* genetic variant was implicated in a high risk of salivary gland cancers, TCs, and leukemias [23]. Checkpoint kinase 2 (*CHEK2*) is a gene involved in DNA damage repair, and its mutation was associated with many cancers including those of the breast, colon, kidney, thyroid, and prostate [5,23]. Werner syndrome, caused by *WRN* gene mutation, explains a genetic relationship between soft tissue sarcoma, melanoma, and TC [24]. Cowden syndrome, a hereditary cancer predisposition syndrome due to *PTEN* tumor suppressor gene mutation, is associated with multiple types of cancers, including breast, thyroid, melanoma, endometrial, and colorectal [25]. Studies have also implicated the presence of estrogen receptors in thyroid tissue [16]. There is also a possibility of several undiscovered genetic polymorphisms leading to the increased susceptibility to malignancy. Importantly, our study demonstrated that the diagnosis of two or more primary cancers was associated with higher tumor aggressiveness and higher mortality. Shared genetic susceptibility, environmental risk factors predisposing to cancer, and treatment effects could all be implicated in the development of SPTC.

Breast cancer has been the primary malignancy most commonly associated with SPTC [26,27,28]. While we conclude that the common TC and breast cancer association may be due to high sample size bias, we acknowledge a persistently elevated SIR and the possibility of a bi-directional relationship, potentially explained by the shared hormonal pathways [29,30,31].

To determine whether the molecular signature of primary tumors could influence the development of the SPTC, we analyzed the TCGA and GTEx databases for RNA expression of tumor (n = 5285) and normal (n = 3135) samples. Our analysis determined that eight genes which were frequently differentially expressed across the 13 common cancers available (Supplementary Table S3). Out of the eight genes identified to by differentially expressed across the cancers, six genes were consistently upregulated across all cancer types and included CXCL10 [32,33], UBE2C [34,35], TOP2A [36], PKMYT1 [37], CENPM [38], and KIAA0101 [39] (Supplementary Figure S1). Determination of these mutations in a patient’s primary malignancy could suggest an increased risk of developing SPTC. Interestingly, the genes C15orf48 and MFAP4 showed an inconsistently directional trend across the cancers, suggesting heterogenous signaling and tumorigenesis pathways between cancer types.

The current American Thyroid Association (ATA) guidelines recommend screening patients who are at increased risk of TC development. Interestingly, the panel recommends cervical ultrasound to evaluate the thyroid bed at 6–12 months and then periodically thereon after following primary TC management, but do not recommend specific follow-up periods for patients with SPTC and do not recommend screening at all in patients with familial follicular cell-derived differentiated TC [40]. On the other hand, the United States Preventive Services Task Force recommends only adults with a history of radiation therapy due to previous head and neck cancer, exposure to environmental radiation, immediate family history of TC, and certain genetic conditions to be screened regularly for TC [41]. Specifically, survivors of lymphoma, leukemia, soft tissue tumors, kidney, breast, and uterine cancer are at a persistently elevated risk and should be screened annually for TC. Survivors of salivary gland cancers, melanoma, stomach, lung, colon, ovarian, pancreas, prostate, and bladder cancers should be surveyed for 10 years following initial malignancy diagnosis considering the risk of SPTC development. Survivors of the larynx, oral cavity, orbit, bone, small intestine, and liver cancers should be screened annually for five years post-cancer diagnosis. The recommended follow-up period based on the increased risk of SPTC for common primary malignancies is shown in Table 4. In general, most malignancies should be considered for screening of SPTC within the first three years after diagnosis, after which the increased risk plateaus. Importantly, the most aggressive forms of SPTC develop within the first year, and both patients and clinicians should account for this when considering follow-up.

While the SEER database is retrospective by nature and lacks detailed information regarding treatment such as radiation dosage and type of chemotherapy in primary malignancy, our study analyzed the largest number of SPTC patients to date with extensive follow-up for over four decades. Of note, SPTC diagnosis rates are expected to be slightly elevated due to increased medical surveillance in consequence of initial treatment of primary cancers. Our work could help in the development of better surveillance strategies, though further studies are warranted to define intrinsic and extrinsic exposomes connecting TC with other malignancies. 

## 5. Conclusions

Survivors from various solid and hematological malignancies had an increased risk of SPTC development. Our latency period model may aid clinicians in screening cancer survivors at risk for TC and assist in the development of a follow-up plan according to the latency period attributed to a patient’s primary cancer.

## Figures and Tables

**Table 1:** Standardized Incidence Ratios for SPTC stratified by the site of primary cancer.

	Observed	SIR (95%CI)	AER	Mean Age at SPTC
Oral Cavity and Pharynx	318	3.1 (2.77–3.46)	3.81	59.39
Lip	11	1.3 (0.65–2.32)	0.5	63.32
Tongue	113	3.8 (3.13–4.56)	5.23	59.37
Salivary Gland	61	4.36 (3.34–5.6)	6.55	55.23
Floor of Mouth	15	3.08 (1.72–5.08)	3.83	61.21
Gum and Other Mouth	40	3.05 (2.18–4.16)	3.93	64.19
Nasopharynx	15	2.48 (1.39–4.09)	2.36	59.78
Tonsil	38	1.99 (1.4–2.72)	1.69	58.87
Oropharynx	8	3.21 (1.39–6.33)	3.86	57.91
Hypopharynx	11	3.35 (1.67–6.0)	3.94	61.56
Digestive System	1243	2.01 (1.9–2.12)	1.96	63.13
Esophagus	32	2.2 (1.51–3.11)	2.06	61.16
Stomach	97	2.72 (2.2–3.31)	3.18	61.74
Small Intestine	56	2.83 (2.14–3.68)	3.76	63.57
Colon and Rectum	849	1.85 (1.73–1.98)	1.66	63.85
Colon excluding Rectum	558	1.8 (1.65–1.95)	1.53	64.89
Cecum	111	1.73 (1.43–2.09)	1.38	67.22
Appendix	20	2.24 (1.37–3.46)	2.68	62.87
Ascending Colon	87	1.59 (1.27–1.96)	1.1	65.93
Hepatic Flexure	18	1.26 (0.75–1.99)	0.47	66.08
Transverse Colon	54	2.11 (1.59–2.76)	2.1	64.48
Splenic Flexure	16	1.56 (0.89–2.54)	1.06	65.14
Descending Colon	37	1.93 (1.36–2.66)	1.78	59.96
Sigmoid Colon	203	1.92 (1.67–2.21)	1.83	64.72
Rectum and Rectosigmoid Junction	291	1.98 (1.76–2.22)	1.93	61.86
Anal Canal and Anorectum	34	1.57 (1.09–2.19)	1.35	62.85
Liver and Intrahepatic Bile Duct	58	2.37 (1.8–3.06)	2.39	63.27
Liver	55	2.42 (1.82–3.15)	2.45	63.34
Intrahepatic Bile Duct	3	1.66 (0.34–4.86)	1.44	61.92
Gallbladder	7	1.55 (0.62–3.19)	1.22	63.37
Other Biliary	17	2.74 (1.59–4.38)	3.35	58.8
Pancreas	69	2.89 (2.25–3.65)	3.87	59.57
Retroperitoneum	11	3.19 (1.59–5.7)	4.05	64.35
Peritoneum and Mesentery	11	2.04 (1.02–3.65)	2.77	52.62
Respiratory System	613	2.44 (2.25–2.64)	2.89	66.24
Nose, Nasal Cavity and Middle Ear	25	4.12 (2.66–6.08)	5.79	61.82
Larynx	103	3.4 (2.77–4.12)	4.06	63.26
Lung and Bronchus	483	2.26 (2.06–2.47)	2.61	67.19
Bones and Joints	30	3.65 (2.46–5.21)	3.59	45.85
Soft Tissue including Heart	138	4.51 (3.79–5.33)	5.94	53.86
Skin excluding Basal and Squamous	695	2.19 (2.03–2.36)	2.52	58.19
Melanoma of the Skin	639	2.15 (1.99–2.32)	2.45	58.09
Other Non-Epithelial Skin	56	2.82 (2.13–3.66)	3.35	59.37
Breast	2305	1.58 (1.51–1.64)	1.65	60.56
Female Genital System	834	1.74 (1.62–1.86)	2.15	58.46
Cervix Uteri	141	1.63 (1.37–1.92)	1.87	50.86
Corpus and Uterus	478	1.73 (1.58–1.89)	2.14	61.43
Ovary	161	1.82 (1.55–2.12)	2.34	56.03
Vagina	13	3.68 (1.96–6.3)	7.01	59.57
Vulva	30	1.67 (1.13–2.39)	1.81	61.23
Male Genital System	1167	1.34 (1.26–1.42)	0.5	68.61
Prostate	1105	1.31 (1.23–1.38)	0.46	69.98
Testis	58	2.69 (2.05–3.48)	1.36	42.44
Penis	3	1.14 (0.24–3.33)	0.2	70.45
Urinary System	826	2.43 (2.26–2.6)	2.57	63.62
Urinary Bladder	247	1.34 (1.18–1.52)	0.59	68.27
Kidney and Renal Pelvis	573	3.77 (3.47–4.1)	5.31	61.48
Ureter	4	1.41 (0.38–3.6)	0.74	70.4
Eye and Orbit	33	3.32 (2.29–4.67)	3.94	62.11
Brain and Other Nervous System	74	2.24 (1.76–2.82)	1.75	45.16
Brain	62	2.2 (1.69–2.83)	1.68	42.5
Cranial Nerves	12	2.48 (1.28–4.33)	2.24	58.86
Lymphoma	716	2.93 (2.72–3.15)	3.64	58.16
Hodgkin Lymphoma	115	3.18 (2.62–3.82)	3.4	41.41
Hodgkin—Nodal	111	3.15 (2.59–3.79)	3.35	41.07
Hodgkin—Extranodal	4	4.25 (1.16–10.8)	5.5	50.81
Non-Hodgkin Lymphoma	601	2.89 (2.66–3.13)	3.69	61.36
NHL—Nodal	382	2.77 (2.5–3.06)	3.46	60.52
NHL—Extranodal	219	3.12 (2.72–3.56)	4.12	62.83
Myeloma	89	1.91 (1.54–2.35)	1.79	63.16
Leukemia	187	1.91 (1.64–2.2)	1.44	59.29
Lymphocytic Leukemia	125	1.96 (1.63–2.33)	1.45	62.23
Acute Lymphocytic Leukemia	13	2.47 (1.32–4.23)	0.68	32.57
Chronic Lymphocytic Leukemia	105	1.95 (1.6–2.36)	1.83	65.89
Myeloid and Monocytic Leukemia	60	1.89 (1.44–2.43)	1.57	53.92
Chronic Myeloid Leukemia	31	1.93 (1.31–2.74)	1.7	58.69
Mesothelioma	9	3.9 (1.78–7.4)	5.33	65.78
Kaposi Sarcoma	6	1.58 (0.58–3.45)	0.61	71.07

SPTC: second primary thyroid cancer, SIR: standardized incidence ratio (observed/expected), CI: confidence interval, AER: absolute excess risk per 10,000, NHL: non-Hodgkin lymphoma. SIR was calculated as the ratio of observed numbers to expected numbers. The number of expected thyroid cancer was estimated based on 5-year age-specific and sex-specific cancer incidence rates for the general population. The AER was calculated by subtracting the expected from the observed number of patients with second primary cancer, then dividing the difference by person-years at risk and multiplying by 10,000.

**Table 2:** Risk of SPTC after another malignancy, stratified by patient characteristics at the diagnosis of primary cancer.

Characteristics	Levels	SIR	95%CI	AER	Z Score
Gender	Male	2.01	1.94–2.08	1.42	Reference
	Female	1.83	1.79–1.88	2.30	−4.24 ***
Age, years	<5	18.03	5.10–46.4	0.95	Reference
	5–24	4.90	4.08–12.0	8.31	−1.22
	25–44	2.26	2.04–2.65	11.1	−1.50
	45–64	1.95	1.87–2.09	9.29	−1.53
	65–84	1.67	1.51–1.79	4.46	−1.55
	85+	1.22	0.97–1.53	0.23	−1.60
Race	White	1.84	1.80–1.88	1.80	Reference
	Black	1.87	1.73–2.02	1.22	0.39
	American Indian/Alaska Native	2.34	1.70–3.13	2.69	1.37
	Asian or Pacific Islander	2.67	2.49–2.86	3.46	8.59 ***
Grade	Well differentiated	1.77	1.67–1.88	1.90	Reference
	Moderately differentiated	1.78	1.71–1.84	1.57	0.16
	Poorly differentiated	1.70	1.63–1.78	1.47	−1.06
	Undifferentiated	1.82	1.59–2.08	1.65	0.37
Laterality	Bilateral	2.07	1.62–2.61	2.76	Reference
	Right-sided	2.01	1.93–2.09	2.46	−0.23
	Left-sided	1.93	1.85–2.01	2.29	−0.55
	Not a paired organ	1.82	1.77–1.87	1.48	Reference
	Midline tumor	5.21	3.09–8.24	8.90	2.58 **
Tumor stage	T0	3.72	1.60–7.32	6.42	Reference
	T1	2.34	1.58–4.92	5.05	−0.82
	T2	2.64	1.12–35.1	2.37	−0.12
	T3	3.09	1.56–77.9	2.66	−0.03
	T4	3.48	1.23–52.0	3.99	−0.02
LN infiltration	N0	2.34	1.63–6.25	6.36	Reference
	N1	2.92	1.90–4.23	4.61	0.44
	N2	3.21	2.10–5.22	5.30	0.61
	N3	3.45	1.46–8.39	7.80	0.52
Distal metastasis	M0	2.50	2.40–2.61	3.52	Reference
	M1	2.34	1.41–4.34	3.20	−0.21
LVI	No	5.89	3.22–9.88	4.04	Reference
	Yes	4.85	1.57–11.3	2.89	−0.35
Clinical stage	I	2.40	1.28–14.19	14.19	Reference
	II	2.36	1.64–14.24	14.24	−0.01
	III	3.49	1.62–15.76	15.76	0.22
	IV	2.98	1.84–5.44	5.44	0.17
Management	Primary site surgery	1.90	1.86–1.93	1.85	Reference
	Radiotherapy	1.78	1.55–2.73	2.15	−0.40
	Chemotherapy	2.10	2.03–2.18	2.58	4.74 ***
	Surgery and radiation	1.86	1.08–5.70	2.23	−0.03

SIR: standardized incidence ratio, CI: confidence interval, AER: absolute excess risk. Excess risk is per 10,000. LVI: lympho-vascular invasion. *p* values for the z score: (**) < 0.01, (***) < 0.001. SIR was calculated as the ratio of observed numbers to expected numbers. The number of expected thyroid cancer was estimated based on 5-year age-specific and sex-specific cancer incidence rates for the general population. The AER was calculated by subtracting the expected from the observed number of patients with second primary cancer, then dividing the difference by person-years at risk and multiplying by 10,000. To compare the SIR of patients with different clinicopathological features, significance (*p*-value) was identified by the estimated z score, which is calculated from SIR and confidence interval values via the following equation: Z = (Y1 − Y2)/SE (Y1 − Y2), where Y1 = ln(OR) for group 1, SE1 = SE (Y1), Y2 = ln(OR) for group 2, SE2 = SE (Y2), and SE (Y1− Y2) = SQRT(SE12 + SE22).

**Table 3:** The risk of SPTC stratified by treatment modalities for primary cancer.

Site	Primary Site Surgery	Radiotherapy	Chemotherapy
All Sites	1.90 (1.86–1.854)	1.78 (1.55–2.73)	2.10 (2.03–2.18)
Oral Cavity and Pharynx	3.10 (2.77–3.46)	3.08 (2.65–3.57)	3.17 (2.62–3.81)
Salivary Gland	4.36 (3.34–5.60)	4.11 (2.77–5.87)	3.74 (1.02–9.57)
Mouth	3.08 (1.72–5.08)	4.55 (1.96–8.96)	7.46 (2.42–17.4)
Tonsil	1.99 (1.40–2.72)	1.59 (1.04–2.32)	1.85 (1.16–2.81)
Oropharynx	3.21 (1.39–6.33)	3.51 (1.41–7.22)	4.41 (1.77–9.08)
Nasopharynx	2.48 (1.39–4.09)	2.86 (1.60–4.72)	2.76 (1.47–4.72)
Hypopharynx	3.35 (1.67–6.00)	3.61 (1.73–6.64)	3.68 (1.59–7.25)
Digestive System	2.01 (1.90–2.12)	2.31 (2.03–2.62)	2.30 (2.12–2.50)
Esophagus	2.20 (1.51–3.11)	2.97 (1.92–4.39)	2.78 (1.82–4.08)
Stomach	2.72 (2.20–3.31)	3.10 (2.03–4.54)	3.01 (2.18–4.05)
Small Intestine	2.83 (2.14–3.68)	NA	3.09 (1.48–5.69)
Colon and Rectum	1.85 (1.73–1.98)	2.23 (1.88–2.64)	2.30 (2.08–2.54)
Liver and Bile Duct	2.37 (1.80–3.06)	4.49 (1.22–11.5)	2.03 (1.27–3.07)
Liver	2.42 (1.82–3.15)	3.46 (0.42–12.5)	2.15 (1.33–3.28)
Intrahepatic Bile Duct	1.66 (0.34–4.86)	6.39 (0.77–23.1)	0.96 (0.02–5.33)
Gallbladder	1.55 (0.62–3.19)	4.40 (1.2–11.27)	3.32 (1.08–7.75)
Pancreas	2.89 (2.25–3.65)	1.55 (0.71–2.93)	2.05 (1.35–2.98)
Respiratory System	2.44 (2.25–2.64)	2.35 (2.05–2.68)	2.16 (1.88–2.47)
Nasal Cavity & Middle Ear	4.12 (2.66–6.08)	4.45 (2.55–7.23)	3.94 (1.45–8.58)
Larynx	3.40 (2.77–4.12)	3.18 (2.50–3.98)	2.89 (1.83–4.34)
Lung and Bronchus	2.26 (2.06–2.47)	1.92 (1.60–2.29)	2.05 (1.77–2.38)
Bones and Joints	3.65 (2.46–5.21)	5.60 (2.42–11.0)	7.33 (4.1–12.09)
Soft Tissue	4.51 (3.79–5.33)	4.48 (3.37–5.83)	6.36 (4.38–8.93)
Skin	2.19 (2.03–2.36)	3.09 (1.77–5.02)	3.57 (1.84–6.23)
Melanoma of the Skin	2.15 (1.99–2.32)	3.76 (1.62–7.40)	3.81 (1.83–7.01)
Other Non-Epithelial Skin	2.82 (2.13–3.66)	2.63 (1.13–5.17)	2.70 (0.33–9.77)
Breast	1.58 (1.51–1.64)	1.52 (1.43–1.61)	1.71 (1.62–1.82)
Female Genital System	1.74 (1.62–1.86)	2.04 (1.66–2.47)	2.05 (1.81–2.32)
Cervix Uteri	1.63 (1.37–1.92)	1.95 (1.34–2.74)	2.20 (1.71–2.78)
Corpus and Uterus	1.72 (1.57–1.89)	1.97 (1.48–2.58)	2.73 (2.16–3.39)
Ovary	1.82 (1.55–2.12)	2.87 (0.59–8.38)	1.63 (1.32–2.00)
Vagina	3.68 (1.96–6.30)	5.18 (1.9–11.27)	4.12 (1.66–8.49)
Vulva	1.67 (1.13–2.39)	2.11 (0.77–4.58)	3.26 (1.19–7.09)
Male Genital System	1.34 (1.26–1.42)	1.35 (1.19–1.52)	2.76 (1.80–4.05)
Prostate	1.31 (1.23–1.38)	1.30 (1.14–1.48)	2.68 (1.28–4.93)
Testis	2.69 (2.05–3.48)	2.46 (1.48–3.84)	2.92 (1.67–4.75)
Urinary System	2.43 (2.26–2.60)	1.65 (0.75–3.13)	1.73 (1.29–2.28)
Urinary Bladder	1.34 (1.18–1.52)	0.96 (0.20–2.82)	1.46 (1.02–2.03)
Kidney and Renal Pelvis	3.77 (3.47–4.10)	2.53 (0.82–5.89)	3.27 (1.83–5.39)
Eye and Orbit	3.32 (2.29–4.67)	2.05 (0.56–5.25)	8.27 (1.7–24.16)
Brain and Nervous System	2.24 (1.76–2.82)	2.26 (1.60–3.10)	2.27 (1.52–3.26)
Lymphoma	2.93 (2.72–3.15)	2.83 (2.40–3.31)	3.04 (2.77–3.33)
Leukemia	1.91 (1.64–2.20)	2.35 (0.95–4.85)	1.94 (1.55–2.41)

Data is presented as Standardized Incidence Rate (95%confidence intervals). SIR was calculated as the ratio of observed numbers to expected numbers. The number of expected thyroid cancer was estimated based on 5-year age-specific and sex-specific cancer incidence rates for the general population. NA: data not available.

**Table 4:** The recommended follow-up period based on the increased risk of SPTC for common primary malignancies.

Recommended Follow-Up	Primary Site of Cancer
5 Years	Liver
10 Years	Colon & Rectum, Lung & Bronchus, Prostate, Melanoma
>10 Years	Breast, Lymphoma, Leukemia

## Data Availability

Data is contained within the article or Supplementary Material.

## Supplementary Materials

The following supporting information can be downloaded at: https://www.mdpi.com/article/10.3390/biomedicines10081984/s1, Table S1: Characteristics of SPTC patients according to the number of previous primary cancers; Table S2: The latency trend in different cancer sites; Table S3. RNA sequencing expression of tumor and normal samples from The Cancer Genome Atlas (TCGA) and the Genotype Tissue Expression project (GTEx). A total of 5285 tumors and 3135 normal samples were identified and analyzed; Figure S1. Heatmap expression pattern of dysregulated genes among different cancers. The most common 50 dysregulated genes (rows) are ranked in a hierarchical fashion (top to bottom). Frequency of dysregulation is represented along a spectrum, with red and white symbolizing frequent and infrequent gene dysregulation respectively. Eight genes were found to be frequently dysregulated across all 13 cancer types. THCA: Thyroid carcinoma; BRCA: Breast adenocarcinoma; COAD: Colon adenocarcinoma; DLBC: Lymphoid neoplasm diffuse large B-cell lymphoma; GBM: Glioblastoma multiforme; HNSC: Head and neck squamous cell carcinoma; KIRC: Kidney renal clear cell carcinoma. LIHC: Liver hepatocellular carcinoma; PAAD: Pancreatic adenocarcinoma; SKCM; Skin cutaneous melanoma; UCEC: Uterine corpus endometrial carcinoma; LUSC: Lung squamous cell carcinoma; PRAD: Prostate adenocarcinoma.
