# Supplementary material for: Latency Trend Analysis as a Guide to Screening Malignancy Survivors for Second Primary Thyroid Cancer
Source: Biomedicines. 2022 Aug 16;10(8):1984. doi: 10.3390/biomedicines10081984 (PMC9406053; doi:10.3390/biomedicines10081984)
Supplement: Supplementary file 1 [file biomedicines-10-01984-s001.zip › biomedicines-1842233-supplementary.pdf]

Table S1. Characteristics of SPTC patients according to the number of previous primary cancers.

Characteristics	Levels	1 PC	2 PC	≥3 PC	Total
**Gender**	Female	8794 (62.9)	832 (57.7)	104 (52.8)	9730 (62.3)
	Male	5186 (37.1)	611 (42.3)	93 (47.2)	5890 (37.7)
**Age, years**	Mean ± SD	61.8 ± 13.9	66.9 ± 12.7	71.2 ± 11.9	62.4 ± 13.9
	<25y	155 (1.1)	3 (0.2)	2 (1)	160 (1)
	<45y	1458 (10.4)	78 (5.4)	5 (2.5)	1541 (9.9)
	<65y	6251 (44.7)	505 (35)	41 (20.8)	6797 (43.5)
	<85y	5674 (40.6)	771 (53.4)	135 (68.5)	6580 (42.1)
	85y+	442 (3.2)	86 (6)	14 (7.1)	542 (3.5)
**Race**	White	11746 (84)	1241 (86)	170 (86.3)	13157 (84.2)
	Black	918 (6.6)	90 (6.2)	11 (5.6)	1422 (9.1)
	Other	1297 (9.3)	109 (7.6)	16 (8.1)	22 (0.1)
**Histological type**	Papillary	11810 (84.5)	1189 (82.4)	156 (79.2)	13155 (84.2)
	Follicular	1168 (8.4)	129 (8.9)	18 (9.1)	1315 (8.4)
	Medullary	306 (2.2)	30 (2.1)	6 (3)	342 (2.2)
	Anaplastic	150 (1.1)	20 (1.4)	3 (1.5)	173 (1.1)
**Tumor stage**	Tx	300 (2.1)	33 (2.3)	7 (3.6)	340 (2.2)
	T0	34 (0.2)	8 (0.6)	2 (1)	44 (0.3)
	T1	3527 (25.2)	398 (27.6)	53 (26.9)	3978 (25.5)
	T2	791 (5.7)	89 (6.2)	14 (7.1)	894 (5.7)
	T3	1205 (8.6)	130 (9)	23 (11.7)	1358 (8.7)
	T4	300 (2.1)	33 (2.3)	7 (3.6)	340 (2.2)
**LN infiltration**	Nx	225 (1.6)	25 (1.7)	7 (3.6)	257 (1.6)
	N0	4672 (33.4)	524 (36.3)	79 (40.1)	5275 (33.8)
	N1	1363 (9.7)	163 (11.3)	28 (14.2)	1554 (9.9)
**Distal metastasis**	Mx	7720 (55.2)	731 (50.7)	83 (42.1)	8523 (54.6)
	M0	6057 (43.3)	691 (47.9)	113 (57.4)	6861 (43.9)
	M1	203 (1.5)	21 (1.5)	1 (0.5)	225 (1.4)
**Clinical stage**	I	3313 (23.7)	347 (24)	50 (25.4)	3710 (23.8)
	II	586 (4.2)	62 (4.3)	11 (5.6)	659 (4.2)
	III	1169 (8.4)	132 (9.1)	22 (11.2)	1323 (8.5)
	IV	794 (5.7)	115 (8)	17 (8.6)	926 (5.9)
**Primary site surgery**	Negative	1491 (10.7)	210 (14.6)	45 (22.8)	1746 (11.2)
	Positive	12031 (86.1)	1183 (82)	143 (72.6)	13357 (85.5)
**Radiotherapy**	Negative	9010 (64.4)	1005 (69.6)	137 (69.5)	10152 (65)
	Positive	4882 (34.9)	433 (30)	59 (29.9)	5374 (34.4)
**Chemotherapy**	Negative	13667 (97.8)	1420 (98.4)	193 (98)	15280 (97.8)
	Positive	313 (2.2)	23 (1.6)	4 (2)	340 (2.2)
**Vital status**	Alive	9586 (68.6)	881 (61.1)	104 (52.8)	10571 (67.7)
	Died	4394 (31.4)	562 (38.9)	93 (47.2)	5049 (32.3)

Data is represented as a number and percentage or mean and standard deviation (SD). PC: primary cancer, SPTC: second primary thyroid cancer, LN: lymph node.

Table S2. The latency trend in different cancer sites.

Primary cancer site	2-11 months	12-59 months	60-119 months	120+ months
	SIR (LL-UL)	SIR (LL-UL)	SIR (LL-UL)	SIR (LL-UL)
**All Sites**	4.06 (3.91-4.22)*	1.75 (1.7-1.81)*	1.36 (1.30-1.42)*	1.39 (1.29-1.49)*
**Lymphoma**	9.56 (8.49-10.7)*	2.21 (1.94-2.51)*	1.63 (1.36-1.94)*	2.12 (1.64-2.7)*
**Soft Tissue including Heart**	9.13 (6.43-12.6)*	4.08 (3.07-5.33)*	3.24 (2.18-4.62)*	4.19 (2.44-6.71)*
**Kidney and Renal Pelvis**	7.37 (6.25-8.63)*	3.74 (3.30-4.22)*	2.44 (2.01-2.94)*	3.02 (2.21-4.03)*
**Leukemia**	3.43 (2.54-4.54)*	1.40 (1.08-1.79)*	1.89 (1.42-2.47)*	2.03 (1.26-3.11)*
**Breast**	3.38 (3.11-3.67)*	1.52 (1.42-1.61)*	1.13 (1.03-1.23)*	1.29 (1.14-1.47)*
**Uterus**	3.18 (2.61-3.83)*	1.71 (1.49-1.96)*	1.26 (1.03-1.52)*	1.43 (1.05-1.90)*
**Melanoma of the Skin**	5.42 (4.65-6.28)*	2.05 (1.81-2.32)*	1.54 (1.31-1.81)*	1.31 (0.99-1.70)
**Salivary Gland**	12.4 (7.8-18.8)*	3.32 (2.03-5.13)*	3.45 (1.93-5.69)*	2.17 (0.59-5.55)
**Stomach**	5.31 (3.81-7.21)*	2.33 (1.66-3.19)*	1.87 (1.07-3.04)*	0.37 (0.01-2.05)
**Lung and Bronchus**	4.04 (3.54-4.60)*	1.66 (1.42-1.93)*	1.5 (1.16-1.92)*	1.54 (0.91-2.43)
**Colon and Rectum**	3.46 (3.02-3.95)*	1.86 (1.68-2.05)*	1.31 (1.12-1.52)*	1.28 (0.98-1.63)
**Pancreas**	2.42 (1.53-3.63)*	2.89 (1.95-4.12)*	4.12 (2.19-7.05)*	3.58 (0.74-10.5)
**Prostate**	1.65 (1.39-1.94)*	1.25 (1.14-1.37)*	1.34 (1.21-1.48)*	1.11 (0.92-1.32)
**Ovary**	3.22 (2.35-4.31)*	1.48 (1.14-1.9)*	1.87 (1.36-2.52)*	1.09 (0.52-2.0)
**Urinary Bladder**	1.62 (1.16-2.21)*	1.26 (1.03-1.53)*	1.32 (1.03-1.66)*	1.40 (0.93-2.02)
**Larynx**	13.2 (9.99-17.0)*	2.50 (1.74-3.47)*	0.68 (0.25-1.48)	1.30 (0.35-3.32)
**Bones and Joints**	12 (5.96-21.4)*	4.01 (2.13-6.85)*	1.5 (0.41-3.84)	1.44 (0.17-5.21)
**Oral Cavity and Pharynx**	10.7 (9.12-12.5)*	2.41 (1.98-2.90)*	1.27 (0.90-1.74)	0.81 (0.37-1.53)
**Eye and Orbit**	9.47 (4.89-16.5)*	3.56 (2.04-5.79)*	0.67 (0.08-2.43)	2.51 (0.52-7.34)
**Small Intestine**	5.50 (3.14-8.93)*	3.29 (2.24-4.67)*	1.25 (0.5-2.57)	1.09 (0.13-3.93)
**Liver**	4.15 (2.71-6.08)*	1.68 (1.01-2.63)*	1.95 (0.84-3.84)	1.91 (0.23-6.89)
**Esophagus**	4.52 (2.68-7.14)*	1.49 (0.71-2.74)	1.36 (0.37-3.48)	0 (0-4.1)
**Brain**	1.84 (0.95-3.22)	2.26 (1.54-3.21)*	2.29 (1.40-3.53)*	2.74 (1.37-4.9)*

SIR: standardized incidence ratio, UL: upper limit confidence interval, LL: lower limit of the confidence interval. * $p < 0.05$. SIR was calculated as the ratio of observed numbers to expected numbers.

Table S3. RNA sequencing expression of tumor and normal samples from The Cancer Genome Atlas (TCGA) and the Genotype Tissue Expression project (GTEx). A total of 5,285 tumors and 3,135 normal samples were identified and analyzed.

TCGA	Cancer Name	Tumor	Normal	GTEx	Normal
THCA	Thyroid carcinoma	512	59	Thyroid	278
BRCA	Breast adenocarcinoma	1085	112	Breast	179
COAD	Colon adenocarcinoma	275	41	Colon	308
DLBC	Lymphoid neoplasm diffuse large B-cell lymphoma	47	-	Blood	337
GBM	Glioblastoma multiforme	163	-	Brain	207
HNSC	Head and neck squamous cell carcinoma	519	44	-	-
KIRC	Kidney renal clear cell carcinoma	523	72	Kidney	28
LIHC	Liver hepatocellular carcinoma	369	50	Liver	110
PAAD	Pancreatic adenocarcinoma	179	4	Pancreas	167
SKCM	Skin cutaneous melanoma	461	1	Skin	557
PRAD	Prostate adenocarcinoma	492	52	Prostate	100
UCEC	Uterine corpus endometrial carcinoma	174	13	Uterus	78
LUSC	Lung squamous cell carcinoma	486	50	Lung	288
PRAD	Prostate adenocarcinoma	492	52	Prostate	100
		**5285**	**498**		**2637**

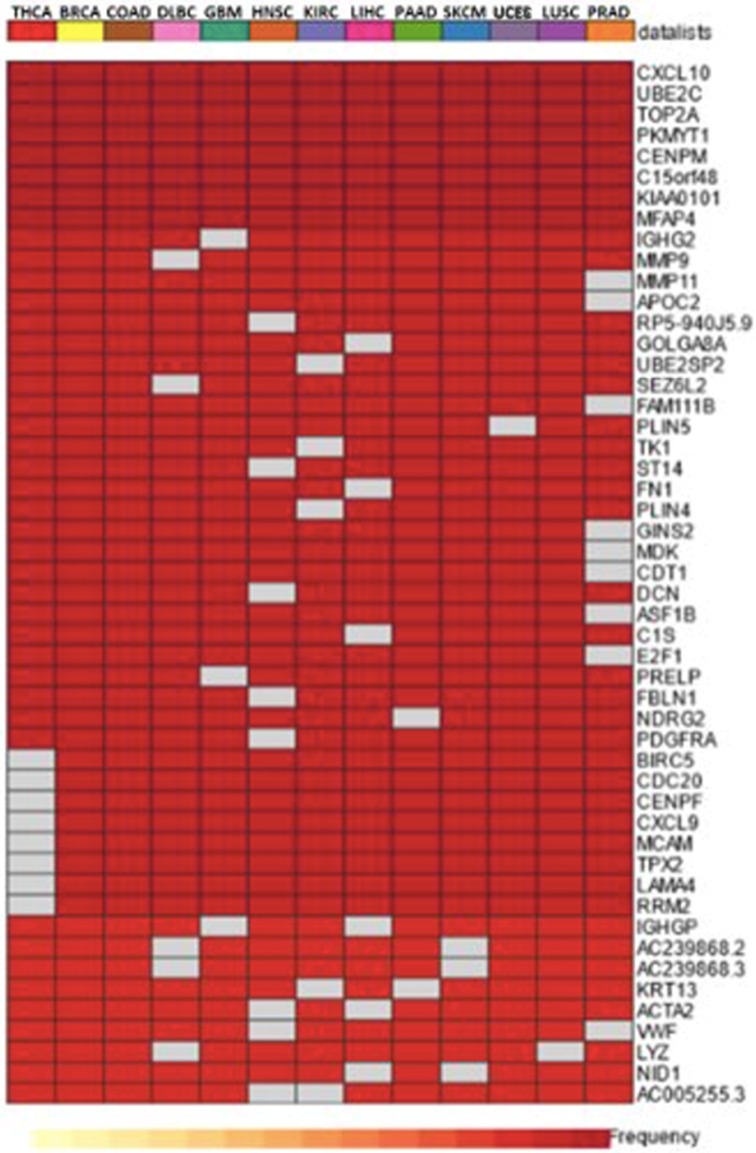

Figure S1. Heatmap expression pattern of dysregulated genes among different cancers. The most common 50 dysregulated genes (rows) are ranked in a hierarchical fashion (top to bottom). Frequency of dysregulation is represented along a spectrum, with red and white symbolizing frequent and infrequent gene dysregulation respectively. Eight genes were found to be frequently dysregulated across all 13 cancer types. THCA: Thyroid carcinoma; BRCA: Breast adenocarcinoma; COAD: Colon adenocarcinoma; DLBC: Lymphoid neoplasm diffuse large B- cell lymphoma; GBM: Glioblastoma multiforme; HNSC: Head and neck squamous cell carcinoma; KIRC: Kidney renal clear cell carcinoma. LIHC: Liver hepatocellular carcinoma; PAAD: Pancreatic adenocarcinoma; SKCM; Skin cutaneous melanoma; UCEC: Uterine corpus endometrial carcinoma; LUSC: Lung squamous cell carcinoma; PRAD: Prostate adenocarcinoma.
